# Resvega Alleviates Hydroquinone-Induced Oxidative Stress in ARPE-19 Cells

**DOI:** 10.3390/ijms21062066

**Published:** 2020-03-17

**Authors:** Niina Bhattarai, Eveliina Korhonen, Maija Toppila, Ali Koskela, Kai Kaarniranta, Yashavanthi Mysore, Anu Kauppinen

**Affiliations:** 1 School of Pharmacy, Faculty of Health Sciences, University of Eastern Finland, 70210 Kuopio, Finland; eveliina.korhonen@uef.fi (E.K.); maija.toppila@uef.fi (M.T.); yashavanthi.mysore@uef.fi (Y.M.); 2 Department of Clinical Chemistry, HUSLAB, Helsinki University Hospital, 00290 Helsinki, Finland; 3 Department of Ophthalmology, Institute of Clinical Medicine, University of Eastern Finland, 70210 Kuopio, Finland; ali.koskela@uef.fi (A.K.); kai.kaarniranta@uef.fi (K.K.); 4 Department of Ophthalmology, Kuopio University Hospital, 70210 Kuopio, Finland

**Keywords:** hydroquinone, Resvega, retinal pigment epithelial cell, ROS, ARPE-19, cell viability, inflammation, antioxidant, NF-κB, NADPH oxidase

## Abstract

Retinal pigment epithelial (RPE) cells maintain homeostasis at the retina and they are under continuous oxidative stress. Cigarette smoke is a prominent environmental risk factor for age-related macular degeneration (AMD), which further increases the oxidant load in retinal tissues. In this study, we measured oxidative stress and inflammatory markers upon cigarette smoke-derived hydroquinone exposure on human ARPE-19 cells. In addition, we studied the effects of commercial Resvega product on hydroquinone-induced oxidative stress. Previously, it was observed that Resvega induces autophagy during impaired protein clearance in ARPE-19 cells, for which it has the potential to alleviate pro-inflammatory pathways. Cell viability was determined while using the lactate dehydrogenase (LDH) and the 3-(4,5-dimethylthiazol-2-yl)-2,5-diphenyltetrazolium bromide (MTT) assays, and the cytokine levels were measured using the enzyme-linked immunosorbent assay (ELISA). Reactive oxygen species (ROS) production were measured using the 2′,7′-dichlorofluorescin diacetate (H_2_DCFDA) probe. Hydroquinone compromised the cell viability and increased ROS production in ARPE-19 cells. Resvega significantly improved cell viability upon hydroquinone exposure and reduced the release of interleukin (IL)-8 and monocytic chemoattractant protein (MCP)-1 from RPE cells. Resvega, N-acetyl-cysteine (NAC) and aminopyrrolidine-2,4-dicarboxylic acid (APDC) alleviated hydroquinone-induced ROS production in RPE cells. Collectively, our results indicate that hydroquinone induces cytotoxicity and increases oxidative stress through NADPH oxidase activity in RPE cells, and resveratrol-containing Resvega products prevent those adverse effects.

## 1. Introduction

The prevalence of degenerative diseases, such as age-related macular degeneration (AMD), increases along with the extension of human life expectancies [1,2]. Currently, it is estimated that AMD affects 170 million people worldwide, which is a considerable cause of severe visual impairment and blindness worldwide [2]. Changes in advanced AMD mostly appear at the macular area resulting in impaired central vision, which makes it difficult e.g., to recognize faces and objects, and eventually to live independently [1,2,3,4]. Retinal pigment epithelium (RPE) cells are primarily degenerated during the pathogenesis of AMD, which subsequently results in functional disruption and eventually the death of photoreceptors [3,4,5].

Cigarette smoke is one of the most important environmental risk factors of AMD, and it is known to damage RPE cells [6]. It promotes the formation of 4-hydroxynonenal (4-HNE), the end product of lipid peroxidation that activates Nucleotide-binding domain, Leucine-rich repeat, and Pyrin domain 3 (NLRP3) inflammasome in RPE cells [6,7]. In addition, it has been shown to trigger vascular endothelial growth factor (VEGF) production resulting neovascularization, a character of exudative (wet) AMD [6,8,9]. Hydroquinone is one component of cigarette smoke [6,10]. It is also included in industrial chemicals and serves as a metabolite of carcinogenic benzene that appears in the petroleum industry [5,11]. Hydroquinone is an aromatic compound that can redox cycle with its own semiquinone radical and produce reactive oxygen species (ROS), such as superoxide, hydrogen peroxide, and hydroxyl radical [12]. Hydroquinone is cytotoxic, immunotoxic, and carcinogenic, as well as immunosuppressive upon prolonged exposure [5,12]. In addition, it increases apoptosis, damages cellular macromolecules, activates cell signaling pathways, such as caspases, and it can be unfavorable for redox-sensitive molecules, such as nuclear factor kappa B (NF-κB) [5,6,11,12,13,14,15]. Hydroquinone has been shown to increase oxidative stress, compromise cell viability, and decrease mitochondrial membrane potential in a concentration-dependent manner in human RPE cells [16,17]. In addition, hydroquinone induces the formation of membrane blebs, which promotes the development of drusen and induces actin protein rearrangement to globular aggregates in RPE cells [18,19,20]. Smokers have increased hydroquinone plasma levels, and hydroquinone probably reaches RPE cells through circulation [21].

Resvega (Laboratoires Théa, Clermont-Ferrand, France) is a commercial product that includes omega-3 fatty acids (EPA 30% *w*/*w*; DHA 15% *w*/*w*; DPA 4% *w*/*w*), trans-resveratrol (2% *w*/*w*), vitamins C (19% *w*/*w*) and E (2% *w*/*w*), minerals (Cu 0.1% *w*/*w*; Zn 1% *w*/*w*), lutein (1% *w*/*w*), and zeaxanthin (0.2% *w*/*w*). It has been previously shown to induce autophagy in human RPE cells [22]. Omega-3 fatty acids are a major component in the product and they appear as a diluent for other components. Omega-3 fatty acids prevail among the fatty acids of photoreceptors, have a protective role in the retina, reduce pro-inflammatory signaling, and prevent apoptosis [23]. In our experiments, the Resvega concentrations used on cell cultures were calculated according to the resveratrol concentration. Resveratrol has a direct ROS-scavenging capacity, but also a property to alleviate oxidative stress through cell signaling pathways [24,25]. It also protects human hepatocyte-derived cells from mitochondria-mediated oxidative stress and promotes the AMP-activated protein kinase (AMPK) pathway, which increases cell survival under stressful conditions [24]. Vitamins C and E directly scavenge oxygen radicals [26]. Copper and zinc are cofactors for superoxide dismutase that converts superoxide radicals to hydrogen peroxide, and catalase again to oxygen. Antioxidants, lutein and zeaxanthin, are not produced by the body and they need to be obtained from food.

In this study, we investigated hydroquinone-induced oxidative stress and the effects of Resvega on it in human ARPE-19 cells. Hydroquinone enhanced oxidative stress by increasing intracellular ROS production, and Resvega showed the potential to alleviate it. Oxidative stress is associated with the pathogenesis of AMD and is known to promote inflammasome activation in human RPE cells [7,27].

## 2. Results

### 2.1. High Concentrations of Hydroquinone Reduce the Viability of ARPE-19 Cells

**Figure 1:**
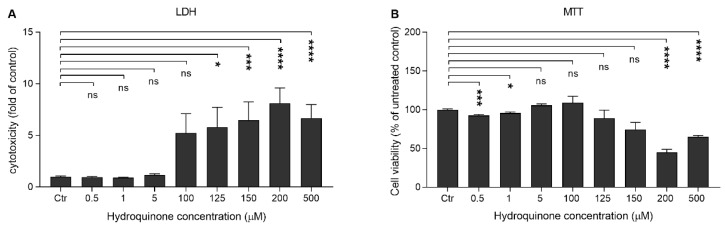
Cell viability of ARPE-19 cells upon hydroquinone exposure. The viability of untreated control cells (Ctr) was set to be 1 in case of lactate dehydrogenase (LDH) (**A**) and 100% in 3-(4,5-dimethylthiazol-2-yl)-2,5-diphenyltetrazolium bromide (MTT) (**B**). Other groups were compared individually to untreated control cells. Results are combined from three independent experiments with 4 parallel samples per group in each experiment and shown as mean ± SEM. * *p* < 0.05, *** *p* < 0.001, **** *p* < 0.0001, ns—not significant, Mann-Whitney U-test.

ARPE-19 cells were exposed to hydroquinone concentrations between 0.5–500 μM for 24 h in order to find the optimal concentration for further studies. Hydroquinone induced cell membrane rupturing at concentrations higher than 100 μM (Figure 1A), whereas cell metabolism was significantly compromised when hydroquinone concentration reached 200 μM (Figure 1B). Increased lactate dehydrogenase (LDH) release conversely correlated with 3-(4,5-dimethylthiazol-2-yl)-2,5-diphenyltetrazolium bromide (MTT) results; while LDH started to increase above the 100 μM hydroquinone concentration, the MTT levels concurrently reduced. According to these results, we selected hydroquinone 125 μM for further experiments. Hydroquinone slightly compromised the cell viability, which still remained at ca. 89%. According to the ISO 10993-5 standard, cells are considered viable when the cell viability is above 80% [28].

### 2.2. Resvega Alleviates Hydroquinone-Induced Cytotoxicity

**Figure 2:**
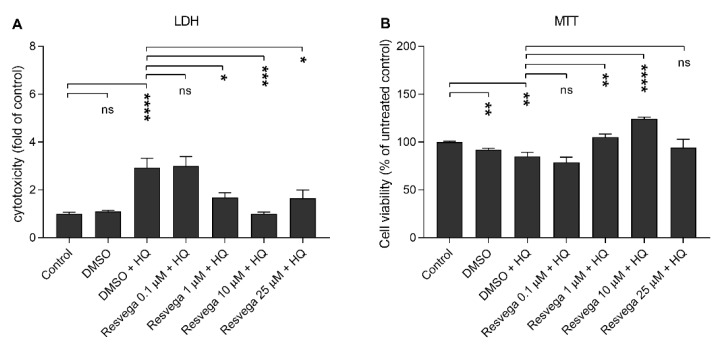
The effect of Resvega (DMSO 0.5% *v*/*v*) on the viability of ARPE-19 cells upon hydroquinone (HQ) exposure. The viability of untreated control cells were set to be 1 in case of LDH (**A**) and 100% in MTT (**B**), to which other groups were compared. The results are combined from three independent experiments with four parallel samples per group in each experiment and shown as mean ± SEM. * *p* < 0.05, ** *p* < 0.01, *** *p* < 0.001, **** *p* < 0.0001, ns—not significant, Mann-Whitney U-test.

We evaluated the ability of Resvega to prevent hydroquinone-induced cytotoxicity using Resvega concentrations 0.1–25 μM on human RPE cells that were exposed to 125 μM hydoquinone. The lowest Resvega concentration (0.1 µM) had no effect on the cell viability when detected using either LDH or MTT assay and compared to hydroquinone-treated cells without Resvega (Figure 2). Instead, 1 µM Resvega increased cell viability, and the effect was the most visible at the 10 μM Resvega concentration. The highest Resvega concentration (25 μM) had no additional effect on the cell viability, although it still prevented LDH release in comparison to cells that were exposed to hydroquinone alone. Additionally, Resvega returned LDH release to the control level and increased viability according to the MTT assay when compared Resvega 10 μM to untreated control cells (Figure 2). We selected Resvega 10 μM for further experiments due to the most promising response to cell viability upon hydroquinone exposure. The used Resvega concentration corresponds to the resveratrol content in the product.

### 2.3. Resvega Reduces IL-8 and MCP-1 Release but Enhances IL-6 in Comparison to RPE Cells Treated with Hydroquinone Only

**Figure 3:**
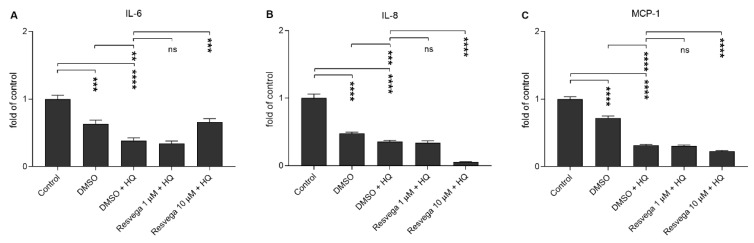
The effect of Resvega (DMSO 0.5% *v*/*v*) to the secretion of inflammatory cytokines IL-6 (**A**), IL-8 (**B**), and MCP-1 (**C**) from hydroquinone (HQ)-exposed RPE cells. The cytokine levels of untreated control cells were set to be 1 and other groups were compared to that. IL-6 concentrations ranged between 1.06–7.33 pg/mL, IL-8 between 2.35–162.25 pg/mL, and MCP-1 between 95.77–1995.88 pg/mL. Results are combined from 3 independent experiments with 4 parallel samples per group in each experiment and shown as mean ± SEM. ** *p* < 0.01, *** *p* < 0.001, **** *p* < 0.0001, ns—not significant, Mann-Whitney U-test.

The effect of Resvega on the production of inflammatory cytokines by human RPE cells was evaluated by measuring the levels of interleukin (IL)-6, IL-8, and monocytic chemoattractant protein (MCP)-1 from cell culture medium samples. Hydroquinone alone reduced the production of all three cytokines when compared to the untreated control cells (Figure 3). Resvega 10 μM further reduced the release of IL-8 and MCP-1 but increased the IL-6 secretion when compared to hydroquinone-treated cells without Resvega (Figure 3). The ability of Resvega to reduce cytokine levels did not result from cell death, since viability assays proved the Resvega 10 μM concentration to be non-toxic to RPE cells (Figure 2).

### 2.4. Hydroquinone Reduces the Activivity of NF-κB and Resvega Increases the Levels of p62/SQSTM1 (p62) Protein

**Figure 4:**
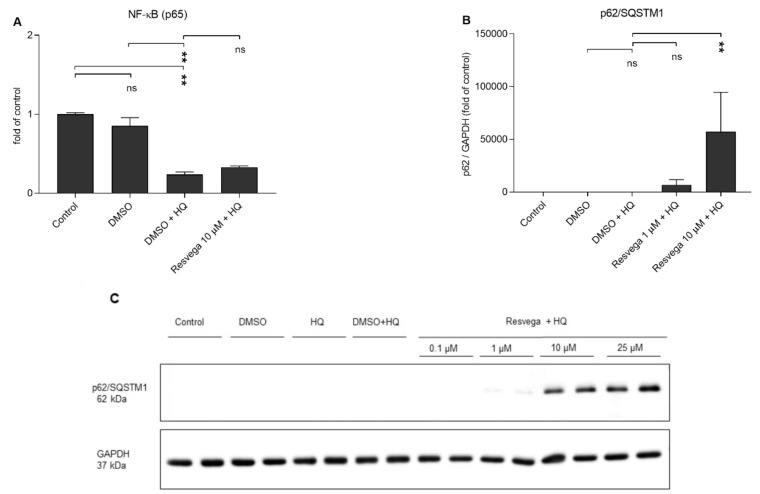
The effect of hydroquinone (HQ 125 μM) and Resvega (0.1–25 μM; DMSO 0.5% *v*/*v*) treatments on the activity of NF-κB (**A**) and the amount of p62/SQSTM1 protein (**B**,**C**). The levels of p62/SQSTM1 protein were normalized to the internal protein control GAPDH (**B**,**C**). The values of untreated control cells were set to be 1 and other groups were compared to that. The results are combined from three independent experiments with two parallel samples per group in each experiment. Results are shown as mean ± SEM. ** *p* < 0.01, ns—not significant, Mann-Whitney U-test.

The effects of hydroquinone and Resvega on the activity of transcription factor nuclear factor kappa B (NF-κB) were evaluated by measuring the DNA binding of the active form of NF-κB (p65). Hydroquinone alone reduced the activity of NF-κB when compared to the DMSO-treated or unexposed control cells, and Resvega had no additional effect (Figure 4A). Resvega at the 10 μM concentration increased the intracellular levels of p62/SQSTM1 protein upon hydroquinone exposure (Figure 4B,C).

### 2.5. Resvega Prevents Hydroquinone-Induced ROS Production

**Figure 5:**
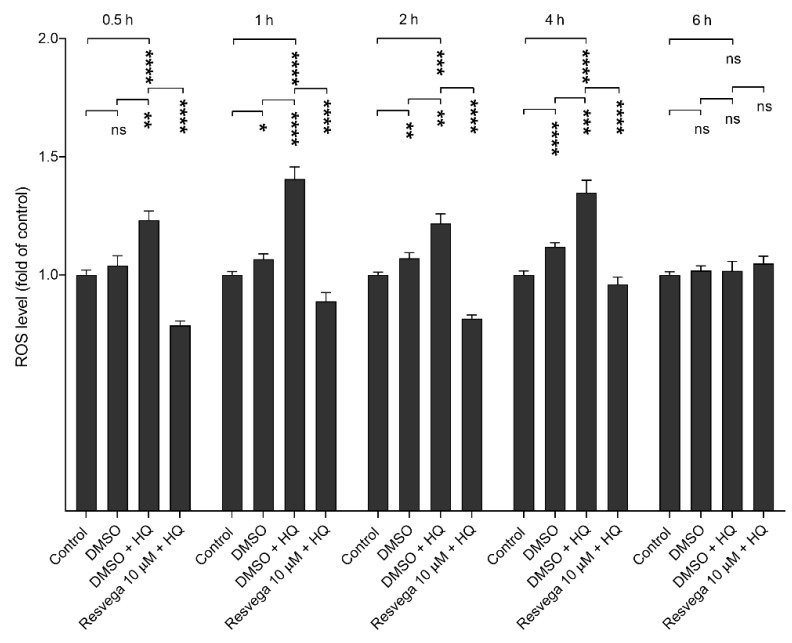
ROS production by hydroquinone (HQ) and the effect of Resvega (DMSO 0.5% *v*/*v*) on it in ARPE-19 cells. ROS levels of untreated control cells were set to be 1 and other groups were compared to that. Results are combined from three independent experiments with six parallel samples per group in each experiment and shown as mean ± SEM. * *p* < 0.05, ** *p* < 0.01, *** *p* < 0.001, **** *p* < 0.0001, ns—not significant, Mann-Whitney U-test.

The propensity of hydroquinone to increase intracellular ROS production and the effect of Resvega on it were evaluated while using the 2′,7′-dichlorofluorescin diacetate (H_2_DCFDA) probe. Hydroquinone significantly increased the ROS production until 4 h, and Resvega significantly reduced it at all time points (Figure 5). At the 6 h time point, hydroquinone did not further increase intracellular ROS production, and the effect of Resvega tailed off concurrently.

### 2.6. Antioxidants NAC and APDC Prevent the Hydroquinone-Induced ROS Production

**Figure 6:**
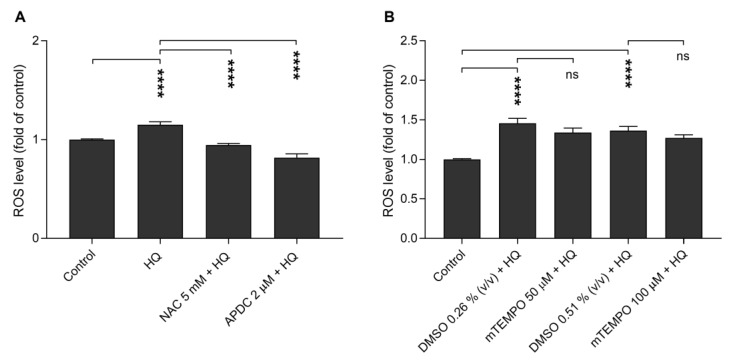
The ability of antioxidants N-acetyl-cysteine (NAC), aminopyrrolidine-2,4-dicarboxylic acid (APDC) (**A**) and mitochondrial-targeted superoxide dismutase (SOD) mimetic (mTEMPO; 50 μM, DMSO 0.26% *v*/*v*; 100 μM, DMSO 0.51% *v*/*v*) (**B**) to prevent hydroquinone (HQ)-induced ROS production. ROS levels of untreated control cells were set to be 1 and other groups were compared to that. The results are combined from three independent experiments with six parallel samples per group in each experiment and shown as mean ± SEM. **** *p* < 0.0001, ns—not significant, Mann-Whitney U-test.

We added different antioxidants to cell cultures in order to investigate the mechanism behind the hydroquinone-induced intracellular ROS production on ARPE-19 cells. N-acetyl-cysteine (NAC, 5 mM) and aminopyrrolidine-2,4-dicarboxylic acid (APDC, 2 μM) significantly prevented the hydroquinone-induced ROS production (Figure 6A), but mitochondrial-targeted superoxide dismutase (SOD) mimetic (mTEMPO, 50 μM and 100μM) had no significant effect (Figure 6B).

## 3. Discussion

Prolonged oxidative stress is a key factor for the initiation of AMD [18]. Oxidative stress is already high during normal functions of RPE cells, such as phagocytosis and the degradation of photoreceptor outer segments, and those cells also experience long-term light exposure [5,26]. Oxidative stress further increases during aging [26]. AMD pathology is associated with dysfunctional intracellular clearance, which results in increased numbers of ROS-producing aged mitochondria and the accumulation of intracellular lipofuscin in the lysosomes of RPE cells as well as extracellular drusen between RPE cells and the Bruch’s membrane [3,4,26]. All of those factors aggravate oxidative stress and induce inflammation that become chronic and contribute to the development of AMD [3,4,26].

In the present study, hydroquinone reduced the release of IL-6, IL-8, and MCP-1 from human RPE cells. This is in line with human lymphocyte studies, where hydroquinone reduced the IL-6, IL-8, and MCP-1 secretion when compared to untreated cells, and mouse macrophage experiments, in which hydroquinone reduced the production of tumor necrosis factor alpha (TNF-α), IL-1β, and IL-6 upon lipopolysaccharide (LPS) exposure [5,29]. The anti-inflammatory effect of hydroquinone can be mediated at least by the prevention of the degradation of inhibitor of kappa B (IκB) protein, which suppresses the activity of transcription factor NF-κB [5,15]. This is in line with our results showing that hydroquinone treatment reduced the activity of NF-κB. Instead of strong promotion of inflammation, hydroquinone increased the production of IL-4 and immunoglobulin E (IgE) in murine lymph node cells [30]. Those factors refer to the type 2 T helper (Th2) cell response that can contribute to the activation of pro-angiogenic M2 macrophages that are associated with choroidal neovascularization and wet AMD [29,31,32,33]. Despite the inhibitory effect of hydroquinone on pro-inflammatory cytokines, Resvega proved its anti-inflammatory potential by further reducing the release of IL-8 and MCP-1, which are chemokines recruiting neutrophils and monocytes, respectively, from the blood stream to the inflamed tissue [34,35]. Instead, Resvega increased the level of IL-6 in hydroquinone-treated RPE cells. The acute phase pro-inflammatory cytokine IL-6 has pleiotropic effects with the ability to promote inflammation and angiogenesis upon pathologic conditions, but it has also shown to be protective in the neural retina [36,37,38]. It has been shown that IL-6 linked autophagy to antioxidative response, and reduced oxidative stress via p62/SQSTM1 and nuclear factor erythroid 2-related factor 2 (NRF2) [39,40,41]. In our previous study, Resvega induced autophagic flux upon declined proteasomal degradation [22]. Protein p62/SQSTM1 alleviates oxidative stress by inducing autophagy and it also activates an antioxidant response [42]. In the present study, Resvega increased the levels of intracellular p62/SQSTM1 protein concurrently with recovery of IL-6 secretion to its control level, but increased IL-6 production was not regulated by NF-κB.

Cigarette smoke destroys the macular area by increasing oxidative stress and suppressing the antioxidative system [9]. Hydroquinone is one component in the cigarette smoke, which is known to induce oxidative stress and apoptosis, and compromise cell viability concentration-dependently in RPE cells [16,21,27,43]. Our results are in line with the observation that hydroquinone-induced cytotoxicity is concentration-dependent and increases oxidative stress in RPE cells [16,17,18].

In the present study, hydroquinone induced ROS production in 30 min in ARPE-19 cells. ROS production remained elevated until 4 h and then returned to the control level by the 6 h time point. The result is in accordance with a study on human lymphocytes, where hydroquinone-induced ROS production started already in 30 min [29]. In our previous experiments, we saw that ROS production also started at early time points (<2h) upon cell-permeable proteasomal inhibitor MG-132 exposure in ARPE-19 cells [42,44]. In contrast to the above-mentioned studies, the research group of Prof. Kenney showed hydroquinone (25–200 μM) to induce intracellular ROS production in ARPE-19 cells as late as 24 h after the exposure [16,17]. In addition, the K562 cells of human chronic myelogenous leukemia expressed increased intracellular ROS levels in a concentration-dependent manner 24 h after the exposure to hydroquinone [14]. That was evident already at low hydroquinone concentrations (≤20 μM), whereas, in mouse monocyte (RAW264.7) cells, low hydroquinone concentrations (≤25 μM) reduced the intracellular ROS levels upon the sodium nitroprusside (SNP) treatment [5,14]. However, the experimental setup was different from our study, since they investigated the antioxidative effects of the non-toxic concentration of hydroquinone under SNP-induced oxidative stress conditions. In addition, conditions, such as medium components and hydroquinone diluent, also have an impact on the effectiveness of hydroquinone [5,16,17,29,45].

Resvega is a mixture of compounds, which all together could have own specific impact, but also combined effects. In addition to resveratrol, Resvega includes other potential protective components. Omega-3 fatty acids possess anti-inflammatory and anti-apoptotic features, and they have been proven protective in retinal diseases [23]. Lutein and zeaxanthin also belong to the retinal antioxidant system [9,26]. Vitamins C and E naturally exist at the retina and they are protective against retinal diseases under oxidative stress conditions [26]. Hutnik et al. (2012) showed that vitamin E enhanced the RPE cell viability under tert-butyl hydroperoxide-induced oxidative stress, and Wei et al. (2014) revealed that vitamin C protects RPE cells from H_2_O_2_-induced oxidative stress [46,47]. Copper (Cu) and zinc (Zn) are both cofactors for superoxide dismutase and they can affect through it to moderate oxidative conditions, and zinc has shown to enhance cell viability and improve mitochondria condition in RPE cells [26,47]. In the present study, we chose to adjust the Resvega concentration according to the concentration of resveratrol.

Resvega reduced the hydroquinone-induced ROS production from 30 min until 4 h. Resveratrol has shown protective effects against mitochondria-mediated ROS production, direct radical scavenging capacity, and the ability to induce protective signaling pathways, such as AMP-activated protein kinase (AMPK) pathway [24,25,48]. It has appeared to be protective and capable of increasing the glutathione levels upon mitochondria-mediated oxidative stress on hepatic cells [24]. In the present study, Resvega appeared to be anti-oxidative under hydroquinone-induced oxidative stress, which was mediated by NADPH oxidase, since APDC declined hydroquinone-induced ROS production, but mTEMPO did not. ROS production declined already with comparatively low APDC concentration, suggesting that principal source of hydroquinone-induced ROS is NADPH oxidase-dependent [44,49]. Even if hydroquinone did not directly induce mitochondria-mediated oxidative stress, there is still possibility for secondary production through the crosstalk between those two ROS production mechanisms [49]. NAC directly functions as an oxygen radical scavenger and it is also a precursor of glutathione, which is an innate cellular antioxidant and maintains redox balance [14]. APDC acts as a glutamate receptor agonist and inhibits NADPH oxidase-dependent ROS production [50]. Generally, quinones have been observed to produce ROS by activating NADPH oxidases [51].

From each cigarette, lungs are exposed to 100 μg of hydroquinone [21,52]. Hydroquinone is reported to absorb rapidly and extensively at least from trachea of animals, and it is widely distributed among tissues [53]. We studied the effects of Resvega on human RPE cells upon hydroquinone-induced cellular stress by adjusting the Resvega concentration according to its resveratrol component. The novelty value of this investigation in comparison to the age-related eye disease studies (AREDS) is that, in addition to other components, Resvega included the resveratrol supplement [54,55]. On the basis of this study, Resvega has protective effects against hydroquinone-induced cytotoxicity and oxidative stress on human RPE cells.

## 4. Materials and Methods

### 4.1. Cell Culture

The ARPE-19 cell line from the American Type Culture Collection (ATCC) was used in all experiments. The cells were cultured in DMEM/F12 (1:1) growth medium (Life Technologies, Paisley, UK) with 2 mM L-glutamine (Life Technologies, Paisley, UK), 100 U/mL penicillin, 100 μg/mL streptomycin (Life Technologies, Grand Island, NY, USA), and 10% fetal bovine serum (GE Healthcare Life Sciences, South Logan, Utah, USA). The cells were grown at +37 °C in humidified conditions with 5% CO_2_ and then passaged two times a week using 0.25% Trypsin-EDTA (Life Technologies, Paisley, UK).

### 4.2. Cell Stimulations and Treatments

For experiments, the cells were seeded onto 12-well plates at the density of 200 000 cells/well and then incubated for three days in serum-containing medium at +37 °C, 5% CO_2_. The confluent cells were washed once using serum-free medium and experiments were performed with same medium. L-glutamine adding was prepared to the medium just before first experiment, and two repetitions were conducted next with the same medium, one experiment after one week. The cells were first exposed to 0.5, 1, 5, 100, 125, 150, 200, and 500 μM hydroquinone (HQ; Sigma-Aldrich, St Louis, MO, USA) and incubated for 24 h at +37 °C, 5% CO_2_ in order to find optimal concentration.

In order to examine the effects of Resvega (Laboratoires Théa, Clermont-Ferrand, France), it was added to cells at concentrations that corresponded to resveratrol concentrations 0.1, 1, 10, or 25 μM. Resvega was added 1 h prior to 125 μM hydroquinone and then incubated for 24 h after the addition of hydroquinone. Resvega contains omega-3 fatty acids (EPA 30% *w*/*w*; DHA 15% *w*/*w*; DPA 4% *w*/*w*), trans-resveratrol (2% *w*/*w*), vitamins C (19% *w*/*w*) and E (2% *w*/*w*), minerals (Cu 0.1% w/w; Zn 1% *w*/*w*), lutein (1% *w*/*w*), and zeaxanthin (0.2% *w*/*w*). Resvega was dissolved in dimethyl sulfoxide (DMSO; Medintech Inc., Corning, Manassas, VA, USA), for which the equal amount of DMSO (0.5% *v*/*v*) was also added to hydroquinone-treated cells.

The cells were seeded onto 96-well plates at 15,000 cells/well in serum-containing medium and incubated for three days for investigating hydroquinone-induced intracellular ROS production. With or without one-hour pre-treatment with Resvega (10 μM), cells were exposed to 125 µM of hydroquinone for 0.5, 1, 2, 4, or 6 h and incubated at +37 °C, 5% CO_2_.

The cells were exposed to hydroquinone for one hour to investigate the mechanism of intracellular ROS production. N-acetyl-cysteine (NAC; 5 mM, Sigma-Aldrich, St. Louis, MO, USA) or mitochondrial-targeted SOD mimetic (mTEMPO; 50 μM, 100 μM, Enzo Life Sciences, Farmingdale, NY, USA) were added one hour before, and aminopyrrolidine-2,4-dicarboxylic acid (APDC; 2μM, Sigma-Aldrich, St. Louis, MO, USA) five minutes before the 125 μM hydroquinone exposure [44,47,56,57]. NAC, mTEMPO, APDC were left on cells concurrently with hydroquinone for 1 h. NAC and APDC were diluted to sterile water and their responses were compared to cells that were only exposed to hydroquinone. mTEMPO was dissolved in DMSO (50 μM, 0.26% *v*/*v*; 100 μM, 0.51% *v*/*v*), for which DMSO was added to some hydroquinone-treated cells and used as controls for the mTEMPO group.

### 4.3. Cell Viability Assays

Cell viability assays were performed to probe the optimal concentration of hydroquinone. Half of the medium (500 μL) was collected after treatments, and then centrifuged (Biofuge Fresco Heraeus Instruments, Newport Pagnell, UK) at 381g for 10 min at +4 °C. Lactate dehydrogenase (LDH) measurement was performed immediately without freezing, and rest of the medium was stored at −20 °C until ELISA measurements. LDH release was analyzed while using a commercial kit (CytoTox96^®^ Non-Radioactive Cytotoxicity Assay, Promega, Madison, WI, USA). The absorbance values were determined using a spectrophotometer (BioTek, ELx808, Microplate reader with the Gen-5 2.04 program; BioTek Instruments Inc, Winooski, VT, USA) at the wavelength of 490 nm.

In addition to LDH measurement, we examined cell viability using the 3-(4,5-dimethylthiazol-2-yl)-2,5-diphenyltetrazolium bromide (MTT) assay (Sigma-Aldrich, St Louis, MO, USA). LDH indicates cellular membrane rupturing, whereas MTT refers to the metabolic activity of cells. MTT assay was performed by adding 25 μL MTT-solution (10 mg/mL in PBS) onto the cells at 12-wells containing 500 µL medium and then incubated for 3 h in dark at +37 °C, 5% CO_2_. Thereafter, the wells were emptied and 1 mL DMSO (Fischer Scientific, Leics, UK) was added to cells and incubated at room temperature for 20 min. After that, 200 μL of DMSO from each well was transferred to a clean 96–well plate and the absorbance values were measured using a spectrophotometer at the wavelength of 560 nm.

### 4.4. Enzyme-Linked Immunosorbent Assay (ELISA)

The levels of cytokines IL-6, IL-8, and MCP-1, as well as the active form of NF-κB (p65) were measured according to the protocols of kit manufacturers. The pro-inflammatory cytokines IL-6 and IL-8 were measured using BD OtpEIA^TM^ Human ELISA Kits (BD Biosciences, San Diego, CA, USA), MCP-1 using the Invitrogen Human CCL2 (MCP-1) kit (Thermo Fisher Scientific, San Diego, CA, USA), and p65 DNA binding using the TransAM NF-κB p65 activation assay kit (Active motif, Carlsbad, CA, USA). The cytokines were determined from cell culture medium samples and NF-kB activity from cell lysates that were prepared by adding 25 μL of the Mammalian protein extraction reagent (M-PER; Thermo Scientific, Rockford, IL, USA) per well on cell culture plates. The cells were incubated in the lysis buffer for five minutes on ice. Cells from two wells were combined in a microtube and centrifuged (Biofuge Fresco Heraeus Instruments, Newport Pagnell, UK) at 16,089× *g* for 10 min, +4 °C. Protein-containing supernatants were collected and the protein levels were measured using the Bradford method. Equal amounts of protein from each sample were used in the ELISA assay. Absorbance values were measured using a microplate reader (Bio-Rad Model 550 with the Microplate Manager 5.2 program; Bio-Rad Laboratories Inc, Hercules, CA, USA) at the wavelength 450 nm with correction at 655 nm.

### 4.5. Western Blot

The intracellular p62/SQSTM1 protein levels were measured using the western blot method. Protein samples (10 μg) were separated using 15% SDS-PAGE gel and transferred to the nitrocellulose membrane (GE Healthcare, Little Chalfont, Buckinghamshire, UK). The membrane was blocked using 3% milk in 0.3% tween PBS for 1.5 h at room temperature. The primary antibody to p62/SQSTM1 (sc-28359, Santa Cruz Biotechnology Inc., Santa Cruz, CA, USA) was diluted 1:1000 with 0.5% BSA in 0.3% tween PBS and then incubated overnight at +4 °C. The membrane was washed with 0.3% Tween PBS 3 × 5 min, and treated with secondary anti-mouse antibody (NA931, GE Healthcare) diluted 1:10,000 with 3% milk in 0.3% Tween PBS for 2 h at room temperature. The primary antibody to the internal protein control glyceraldehyde 3-phosphate dehydrogenase (GAPDH; ab8245, Abcam, Cambridge, UK) was diluted 1:15,000 with 0.1% tween PBS and then incubated on the membrane for 2 h at room temperature. The membrane was washed with 0.1% tween PBS 3× 5 min, and secondary anti-mouse antibody (NA931, GE Healthcare) was diluted 1:12,000 with 0.1% tween PBS, and then incubated on the membrane for 1 h at room temperature. After secondary antibody treatments, the membrane was washed as previously. Protein-antibody complexes were detected using the chemiluminescent method with substrate (Millipore, Billerica, MA, USA) and the ImageQuant RT ECL (GE Healthcare, Little Chalfont, UK). The protein intensities were quantified using the ImageJ software.

### 4.6. ROS Detection

After cell exposures, medium was removed from the cell culture plates and the wells were washed once with serum-free medium. Thereafter, 5 μM 2′,7′-dichlorofluorescin diacetate (H2DCFDA; Molecular probes, Life technologies, Eugene, OR, USA) was added and the cells were incubated for one hour at +37 °C. The cells were washed twice with Dulbecco’s Phosphate Buffered Saline (DPBS; Life Technologies, Paisley, UK), 100 μL DPBS was added onto the wells, and fluorescence intensity (excitation = 488 nm; emission = 528 nm) was measured using a BioTek Cytation3 imaging reader with Gen-5 3.03 program (BioTek, Instruments Inc, Winooski, VT, USA). The untreated controls and DMSO-treated (0.5% *v*/*v*) cells were used as negative controls.

### 4.7. Statistical Analysis

Statistical analyzes were performed using the GraphPad Prism program 7.04 (Graphpad Software, San Diego, CA, USA). The differences between groups were analyzed using the Mann-Whitney U-test. The results were considered to be statistically significant at *p*-values that were lower than 0.05. The results are shown as means ± standard error of means (SEM).

## 5. Conclusions

Resvega significantly protected human RPE cells from hydroquinone-induced cytotoxicity and oxidative stress. The data provide novel information about the cytoprotective potential of Resvega. This warrants for further studies and needs more detailed examination of the cellular route and dosage of Resvega also *in vivo* usage.
